# Wilms' tumor gene 1 silencing inhibits proliferation of human osteosarcoma MG-63 cell line by cell cycle arrest and apoptosis activation

**DOI:** 10.18632/oncotarget.14715

**Published:** 2017-01-18

**Authors:** Adriana Carol Eleonora Graziano, Venera Cardile, Rosanna Avola, Nunzio Vicario, Carmela Parenti, Lucia Salvatorelli, Gaetano Magro, Rosalba Parenti

**Affiliations:** ^1^ Department of Biomedical and Biotechnological Sciences, Physiology Section, University of Catania, 95125 Catania, Italy; ^2^ Department of Drug Sciences, Pharmacology and Toxicology Section, University of Catania, 95125 Catania, Italy; ^3^ Department G.F. Ingrassia, Azienda Ospedaliero-Universitaria “Policlinico-Vittorio Emanuele” Anatomic Pathology, University of Catania, 95125 Catania, Italy

**Keywords:** WT1, apoptosis, osteosarcoma, oncogene, cell cycle

## Abstract

Wilms’ tumor gene 1 (WT1) plays complex roles in tumorigenesis, acting as tumor suppressor gene or an oncogene depending on the cellular context. A high WT1 expression level was described in various types of human bone and soft-tissue sarcomas, including osteosarcoma (OS), but its function in carcinogenesis is not yet well understood. This study investigated WT1 both in human OS tissues and in human OS MG-63 cell line in which WT1 gene is up-regulated. The results demonstrated that WT1 is expressed in 50% of human OS cases. WT1-silenced MG-63 cells showed deregulation of proteins of cell cycle and down-regulation of PI3K/AKT pathway. Induction of apoptotic programme was also established by activation of caspase-3 and increase of Bax/Bcl2 ratio and p53 protein. This study provided new findings on role of WT1 and indicated an association between WT1 expression, cell cycle and apoptotic machinery. In conclusion, WT1 acts as a tumour promoter in osteosarcoma and it could be a potential therapeutic target.

## INTRODUCTION

Osteosarcoma (OS) is a primary malignant cancer of bone affecting predominantly the children and the young [1]. While therapy for localised OS at onset is on track, a number of OS cases have been observed to be resistant to currently used therapies, leading to disease recurrence and lung metastases [2, 3].

During the last few years, the molecular analysis provided new information about numerous genes and pathways that were associated with OS and its clinical progression. Thus, what is becoming clear is that OS is a contextual attribute of distinct patterns of interactions between multiple genes [4]. However, it is yet far to cracking the molecular picture of this neoplasm. Moreover, the presence of “metastatic heterogeneity” characterizing the wide spectrum of patient survival [2] makes high expectations of identifying new molecular markers for improving OS therapeutical approaches.

The Wilms’ tumour 1 (WT1) gene, located at chromosome 11p13, encodes a zinc transcription factor firstly identified as a tumour suppressor gene in nephroblastoma or Wilms’ tumour, a pediatric kidney cancer [5–7]. A combination of many different protein isoforms, likely explains different and apparently opposite molecular and biochemical functions of WT1 through the exhibition of complex activation programs [8–11]. Thus, WT1 plays a very broad field of action ranging from physiological processes including cell growth/development [12, 13] and embryogenesis [14–19], but also ongoing physiological processes throughout life [20] to a variety of disease states properly linked to control of the mesenchymal-epithelial balance of cells [21]. In particular, WT1 shows a complex role in tumorigenesis acting as both a tumour suppressor gene and an oncogene, or even a biphasic functional actor depending on the operating context [22, 13]. Analyses of WT1 expression in different human cancers already indicate the involvement of this transcription factor in tumour development and progression [23, 13]. Increased WT1 expression was described in carcinomas of the lung [24], breast [25], colon [26], pancreas [27], and in desmoid tumors [28], hematopoietic system tumours [29, 30], myofibroblastoma [31], melanoma [32], brain tumours [33–36]. An increasing number of results strongly indicate that WT1 gene is also involved in tumorigenesis of various types of human bone and soft-tissue sarcomas [23, 37–43]. High levels of WT1 expression was associated to the high grade of proliferation of cancer cells in the examined sarcoma tissue samples [37]. In particular, WT1 seems to be associated with very poor survival of patients with osteogenic sarcoma metastasis [2] even if WT1 expression activation would appear linked to metastatic heterogeneity scheme which governs OS.

In any case, the role of WT1 in OS remains unclear. Thus, the inhibition of WT1 level by specific RNA interference (RNAi) in established OS cell line may be helpful for a better understanding of the range of action of this gene and for investigating its role in the pathogenesis of these tumors.

In this study, we analyzed WT1 expression profile in different human OS tissues and in human OS MG-63 cell line, in which WT1 gene is naturally up-regulated. The aim of using an *in vitro* system was addressed to investigate whether WT1 silencing by RNAi is capable to alter cell cycle progression, cell proliferation and cell transformation. Our WT1 RNAi results indicated a mechanistic and molecular association between WT1 expression and both cell cycle and apoptotic machinery, influencing different key points of signaling pathways.

## RESULTS

### WT1 expression profile in human osteosarcoma tissues

**Figure 1:**
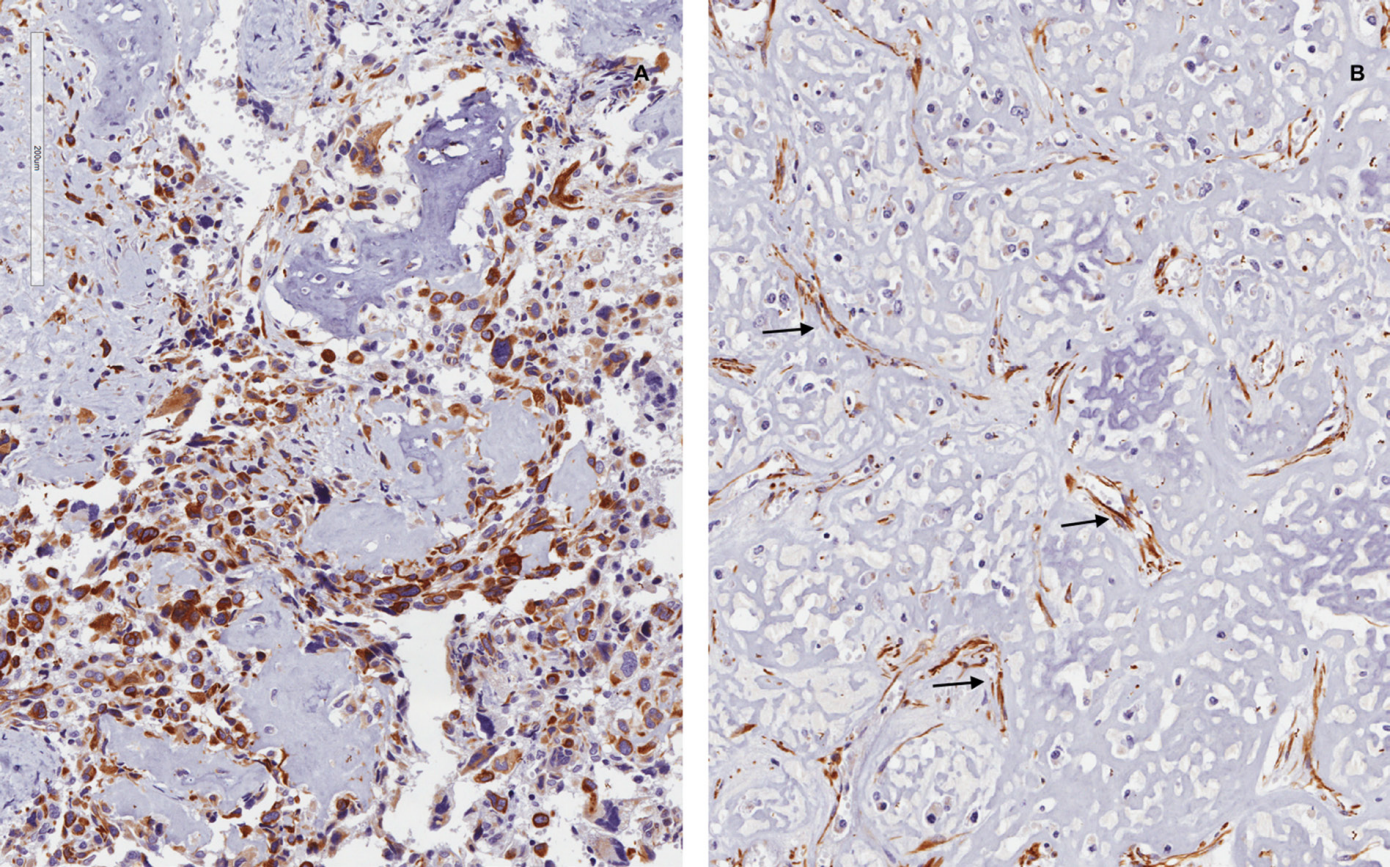
WT1 expression in primary OS of the bone Two cases of primary OS of the bone. Neoplastic cells may variably express WT1. In A there is a diffuse cytoplasmic staining, while no immunoreactivity can be appreciated in B (antibody 6FH2 antibody). Endothelial cells stained with WT1 (cytoplasmic localization) served as internal control (B, black arrows). No nuclear staining for WT1 has been obtained.

Six cases of conventional high-grade osteogenic sarcoma were screened to verify WT1 expression and the protein was expressed exclusively in three cases. Immunostaining was obtained only by using WT1 antibody against N-terminus (clone 6F-H2) and it was almost restricted to cytoplasm of neoplastic cells. Staining intensity and extension were strong and diffuse, respectively (Table 1). No nuclear staining was obtained using both antibodies. Endothelial cells of intra-tumoral blood vessels were stained and served as internal control (Figure 1).

**Table 1:** Correlation between immunohistochemical detection of WT1 and specimens of each patient-derived OS tissue

Tumor type	Sex (M-F)	Age	Polyclonal (C-19) Score°		Monoclonal (6F-H2) Score°	
			Nucleus	Cytoplasm	Nucleus	Cytoplasm
osteogenic sarcoma	M	9	−	−	−	+++
	F	13	−	−	−	+++
	F	14	−	−	−	−
	M	23	−	−	−	−
	F	64	−	−	−	−
	F	68	−	−	−	+++

°Score symbols: − = negative staining; + = focal staining; ++ = heterogeneous staining; +++ = diffuse staining.

### WT1 expression profile in MG-63 cell line

Among the commercially available human OS cell lines, MG-63 cells were selected for this study because an enrichment of WT1 gene has been already reported in these cells [44]; moreover, previous studies have demonstrated that MG-63 cells line has the typical complex genomic structure that characterizes patient derived OS [45]. In addition, we detected WT1 expression in different OS cell lines and only MG-63 reported high and stable level of this protein (Supplementary Figure 1). Under optic microscope, MG-63 cells revealed an oval to spindle-shaped morphology, without branching cell processes and were positively labelled for MMP-9 and Collagen-X, as already reported [46].

**Figure 2:**
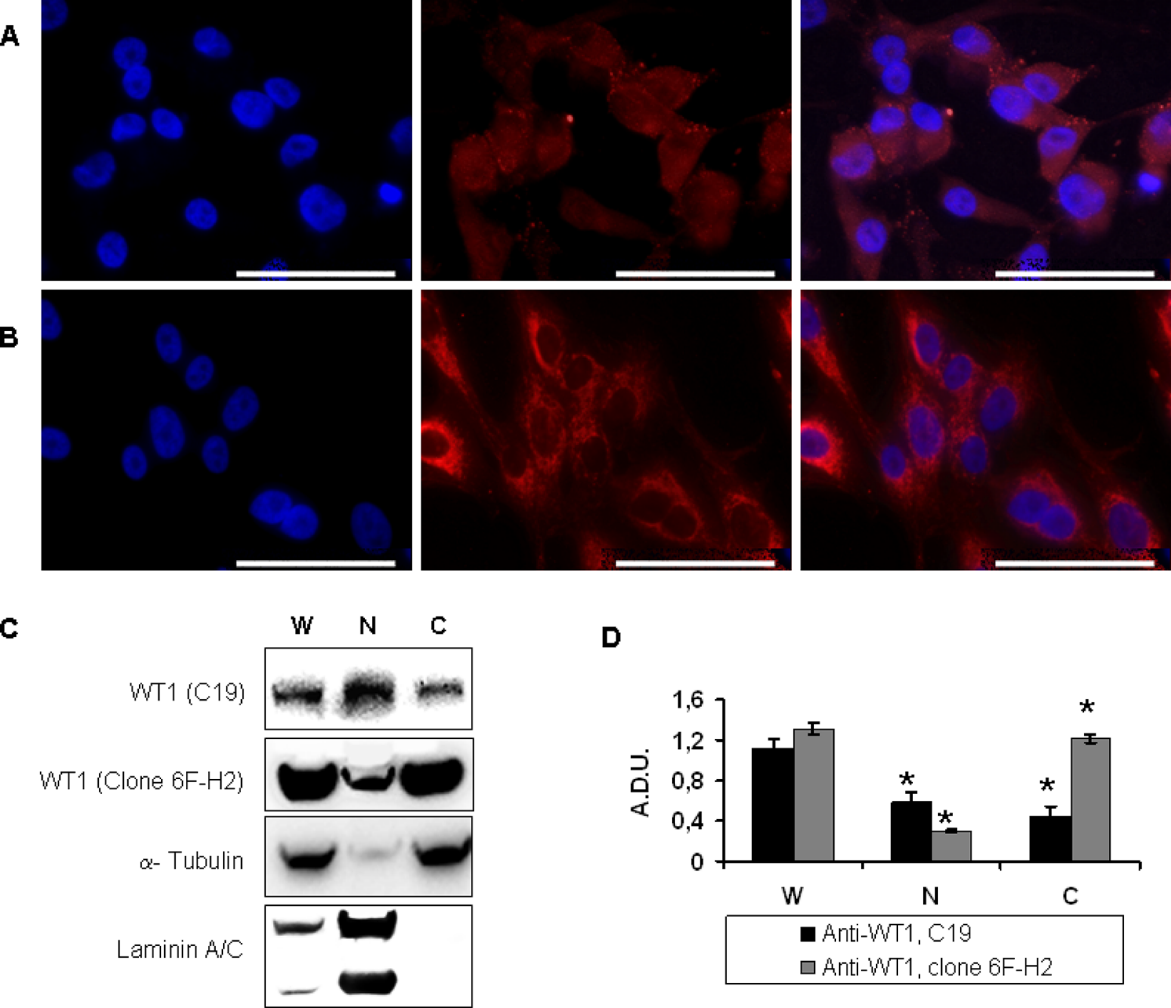
WT1 expression in MG-63 cells determined by immunocytochemistry and Western blot Immunofluorescence positive cells to C-19 WT1 **(A)** and 6F-H2 **(B)** antibodies are merged with their own DAPI-stained nuclei (blue). Scale bars: 50 μm. **(C)** Whole cell lysate (W), cytosolic **(C)**, and nuclear (N) fractions were immunoblotted with C-19 and 6F-H2 WT1 antibodies, respectively. **(D)** Results of three independent immunoblots were expressed as arbitrary densitometric units (A.D.U.) ± SEM (*n* = 3; *P < 0.05 compared to whole cell lysate).

In order to investigate the expression and the localization of WT1, immunocytochemistry was performed by using two antibodies directed against the C-terminal (C-19, sc-192) or the N-terminal (clone 6F-H2) portion of the WT1 molecule, respectively. The results revealed that WT1 protein is expressed in both the nucleus and in the cytoplasmic area around the nucleus, even if the 6F-H2 antibody produced a stronger and more homogeneous reactivity (Figure 2B) in the cytoplasmic area around the nucleus compared to C-19 antibody (Figure 2A).

A deeper investigation of WT1 intracellular localization was performed by Western blot analysis on separated fractions to distinguish the nuclear from the cytoplasmic one, using total cellular lysate as control. Results revealed that WT1 was located in both compartments (Figure 2C) more evidenced by C-19 antibody respect to 6F-H2 antibody that revealed cytoplasmic fraction, prevalently (Figure 2D).

### WT1 siRNA interfered WT1 expression in MG-63 cells

**Figure 3:**
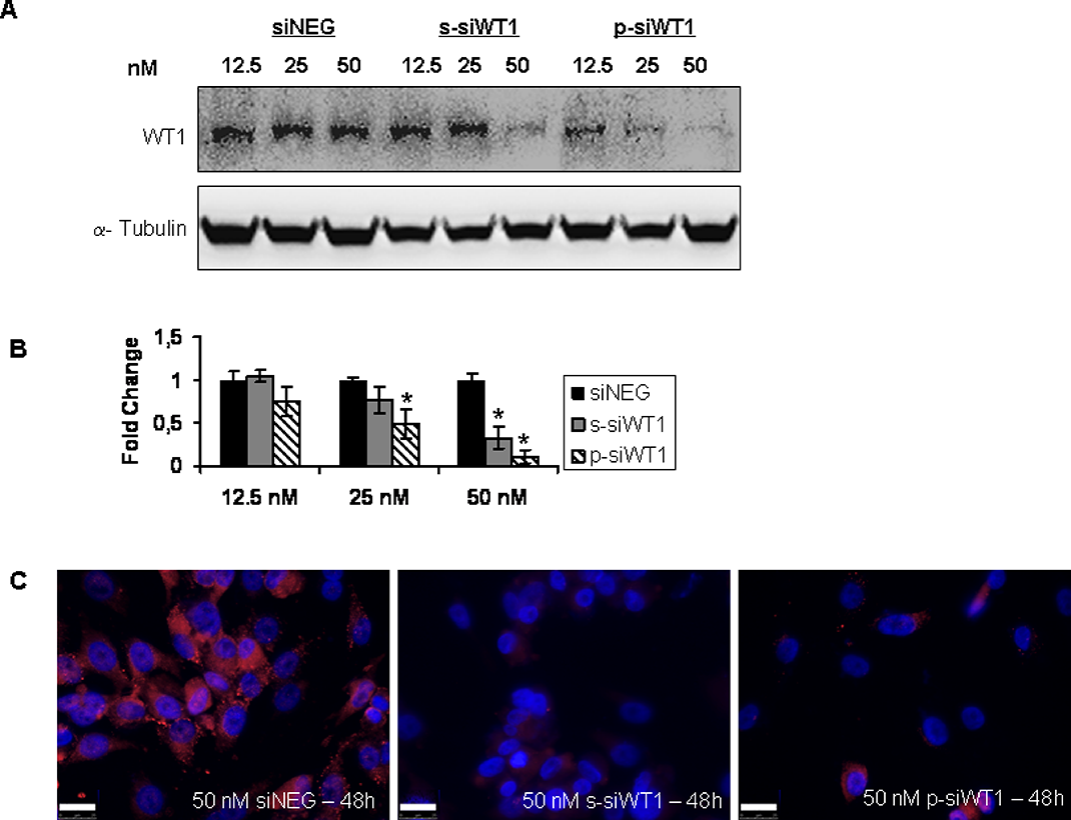
WT1 siRNA interfered WT1 expression in MG-63 cells **(A)** Representative immunoblotting of WT1 in siNEG or siWT1 MG63 cells. **(B)** Results of three independent immunoblots are represented as fold change of WT1 expression respect to each siNEG (*n* = 3; *P < 0.05 compared to respective siNEG group). **(C)** Images of WT1 immunofluorescence in 50 nM siNEG, s-siWT1 and p-siWT1 MG63 cells. Scale bars: 25 μm.

MG-63 cells were transfected with 12.5, 25 and 50 nM siRNA against WT1. The efficiency of transfection was evaluated by fluorescently labeled siRNA (Qiagen) and resulted to be no higher than 70% (data not shown). The transfections were conducted by using a single siRNA (s-siWT1), a pool of three different siRNA (p-siWT1), or a scrambled control (siNEG) for 48 hours. The s-siWT1 was applied in order to exclude off-target effects. MG-63 protein was detected both in the control group (Figure 2C) and the siNEG group (Figure 3A), and no significant difference was observed between the two groups, demonstrating that the negative control did not alter WT1 expression in MG-63 cells. After 48 hours treatment, the expression of WT1 protein was significantly inhibited in the s-siWT1 group at 50 nM and in the p-siWT1 ones at 12.5, 25 and 50 nM (Figure 3A). In this latter group, the interference effect was more pronounced at 50 nM, as revealed both by Western blot (Figure 3B) and by immunocytochemistry (Figure 3C).

### WT1 silencing blocked MG-63 cells proliferation *in vitro*

**Figure 4:**
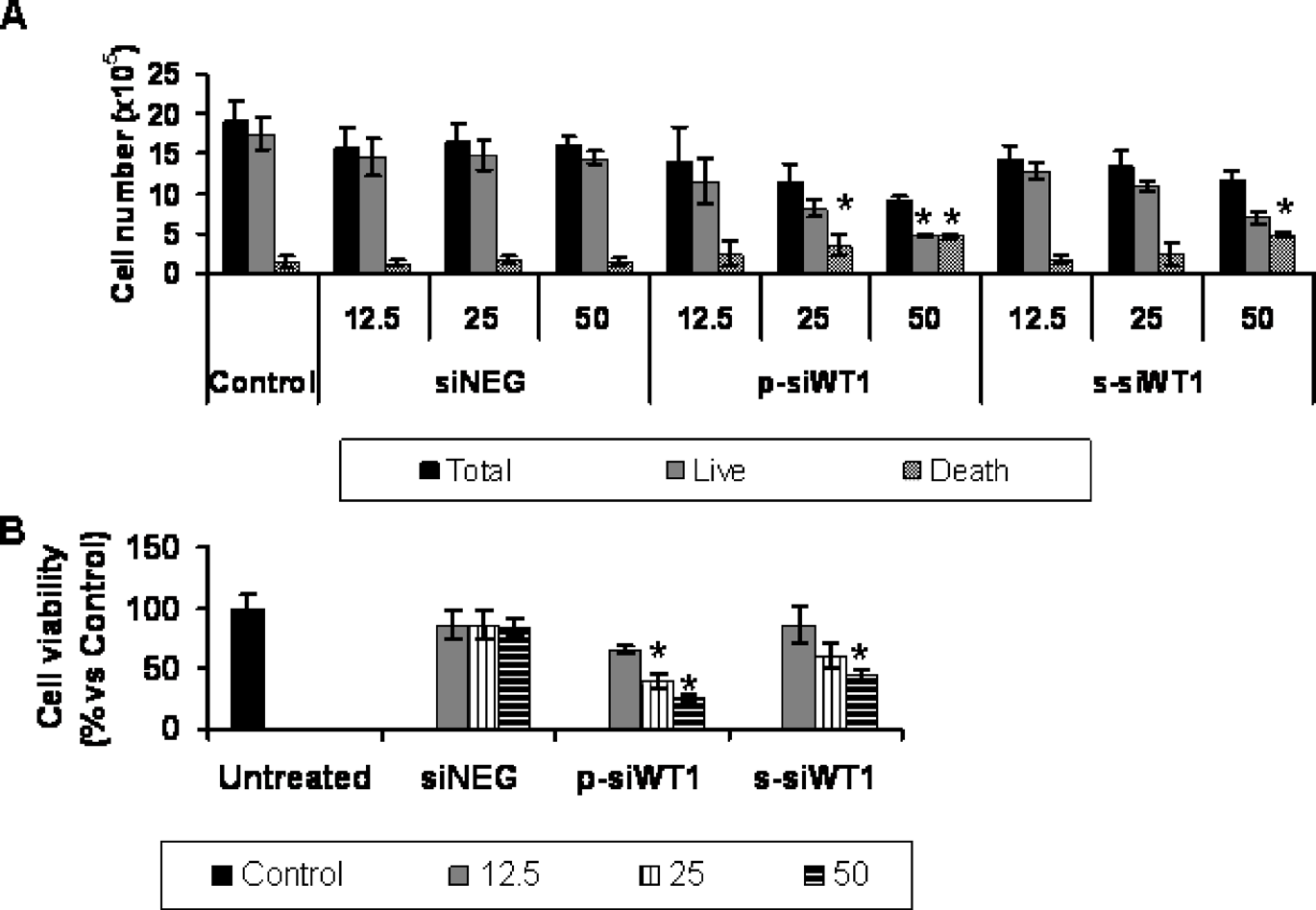
WT1 is involved in proliferation and viability of MG-63 cells in vitro **(A)** Effects of 12.5 nM, 25 nM and 50 nM p-siWT1 and s-siWT1 on MG63 cell proliferation at 48 hours. The total viable cell number was assessed by trypan blue exclusion (*n* = 3; *P < 0.05 compared to respective siNEG group). **(B)** Viability of MG63 cells treated with 12.5 nM, 25 nM and 50 nM siNEG, p-siWT1 and s-siWT1 by MTT assay. Data are reported as percentage ± SEM respect to controls (*n* = 3; *P < 0.05 compared to respective siNEG group).

In physiological condition, MG-63 showed high rate of proliferation with a cell population already doubled in culture to 26 hours. WT1 silencing (siWT1) experiments were performed to determine the effects on cell proliferation using siNEG as a control. Respect to untreated cells, the siNEG treatment for 48 hours induced a cell number decrease up to 20% (Figure 4A), while in si-WT1 cells, the counts underlined a loss of population doubling that ranged from low to intense levels, according to the interference effect resulted by the applied dose of silencing agent. In particular, compared to each siNEG, the cell number started to decrease (Figure 4A), although at low and not significant level, with 12.5 nM siWT1 after 48 hours treatment (p-siWT1: 11 ± 3%; s-siWT1: 8 ± 2%). 25 nM siWT1 induced a reduction of total cell in culture up to 30 ± 4% and 18 ± 4% when treated with p-siWT1 and s-siWT1, respectively (Figure 4A). The effect of 50 nM siRNA was more evident: the s-siWT1 still inhibited the increase of total cell number, whereas the p-siWT1 was able to halve significantly the total cell number respect to 50 nM siNEG (p-siWT1: 44 ± 5%; s-siWT1: 32 ± 5%). Considering that the parameter of total cell number should be too generic because it could reflect a reduction of cell populating doubling or the induction of cell death, the cells were counted as live and death by trypan blue staining (Figure 4A). Each siNEG treatment resulted in a basal appearance of cell death, quantified as 8 ± 1% versus the count of total cells, whereas the presence of silencing RNA induced a reduction of cell proliferation and an induction of cell death. Compared to total cells, the blue staining reached the 17 ± 2, 29 ± 5, and 49 ± 9% for p-siWT1 and the 11 ± 3, 17 ± 9, and 40 ± 3% for s-siWT1 at 12.5, 25 and 50 nM, respectively. MTT assay was also performed to measure the mitochondrial activity. Results reported a dose-dependent decrease of MTT conversion into formazan, related to silencing efficiency (Figure 4B), suggesting to analyze not only cell cycle markers, but also pathways of cell survival and death.

### WT1 silencing altered cell cycle of MG-63 by down-regulating Cyclin D1 and p-pRb Proteins

**Figure 5:**
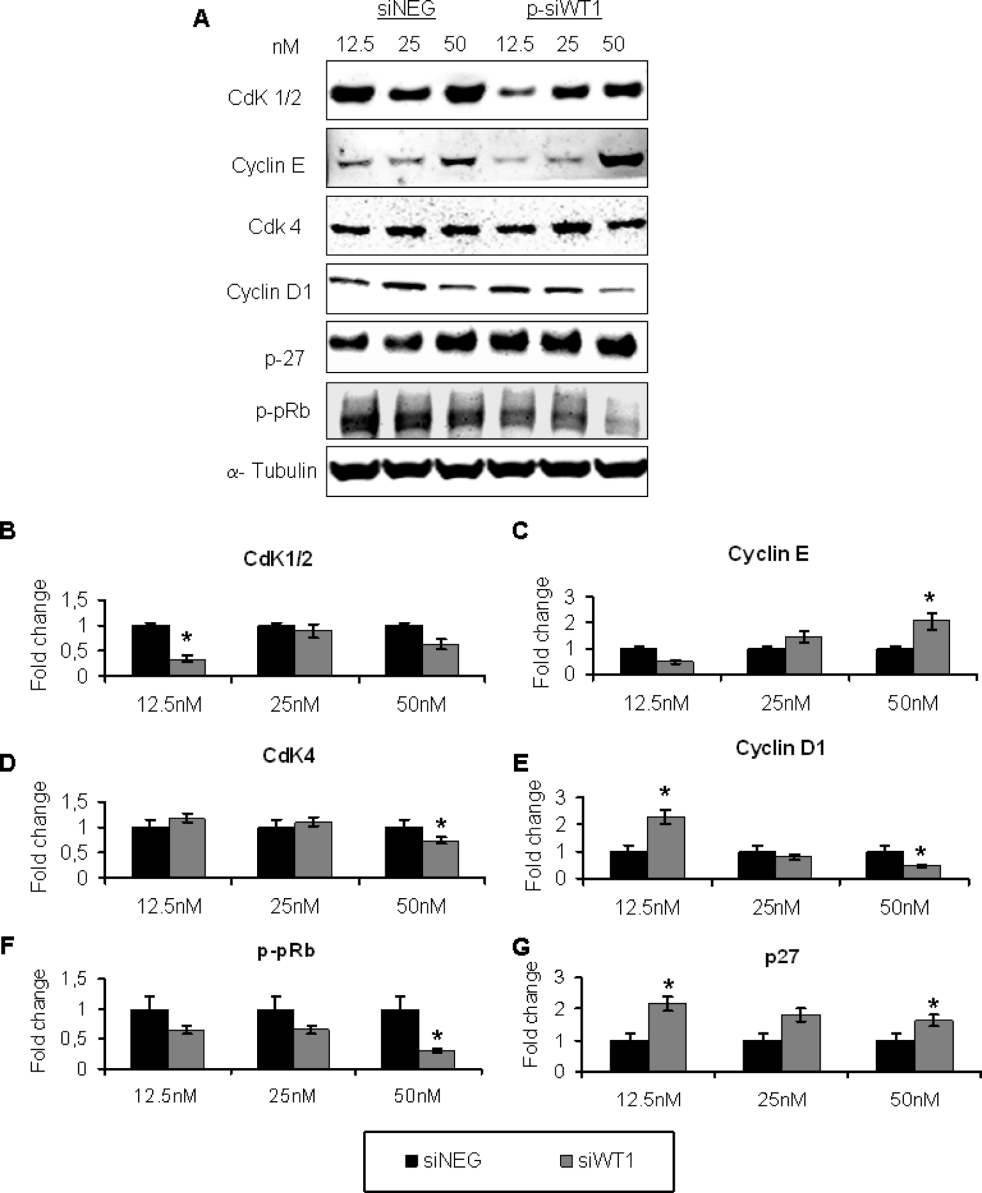
siWT1 is accompanied by changes in cell cycle proteins **(A)** Representative immunoblotting of CdK1/2, Cyclin D1, CdK4, Cyclin E, p27 and p-pRb in control siNEG or siWT1 MG-63 cells. The histograms represent the fold change of CdK1/2 **(B)**, Cyclin E **(C)**, CdK4 **(D)**, Cyclin D1 **(E)**, p-pRb **(F)** and p27 **(G)** expression in WT1-silenced-MG-63 respect to each siNEG group (*n* = 3; *P < 0.05 compared to respective siNEG group).

In order to determine whether the cell proliferation block of WT1-silenced MG-63 was accompanied by changes in proteins involved in cell cycle regulation, the expression of CdK1/2, cyclin D1, CdK4, cyclin E, p27 and p-pRb proteins were studied (Figure 5A). All these proteins showed an altered expression correlated to the intensity of p-siWT1 interference effect. At lowest p-siWT1 treatments, MG-63 reacted with an increase in cyclin D1 (Figure 5E) and CdK4 (Figure 5D) proteins levels, while cyclin E (Figure 5C) and CdK1/2 (Figure 5B) proteins levels decreased. The phosphorylation of Rb protein was also reduced (Figure 5F), probably as direct consequence of p27 upper-expression respect to siNEG (Figure 5G). The intense loss of WT1 expression, resulting from 25 and 50 nM p-siWT1 treatments, elevated the levels of cyclin E and p27. These events were accompanied by a lower expression of CdK1/2, cyclin D1, CdK4, and p-pRb, suggesting that WT1 functioned through multiple regulators to support cell proliferation and survival.

### WT1 silencing interfered with cell survival pathways of MG-63 cell lines

**Figure 6:**
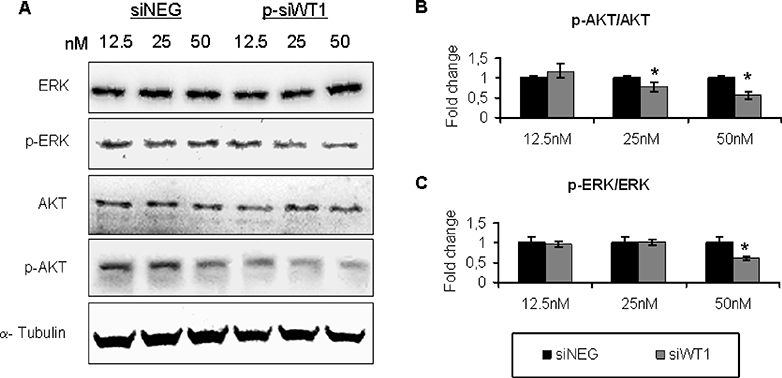
WT1 silencing interfered with phosphorilation of AKT and ERK in MG-63 cell lines **(A)** Representative immunoblotting of pAKT, AKT, pERK, and ERK in MG-63 cells treated with 12.5 nM, 25 nM and 50 nM siNEG or p-siWT1. **(B)** The p-AKT/AKT ratio was reported as fold change in siWT1 groups compared to each siNEG ones (*n* = 3; *P < 0.05 compared to respective siNEG group). **(C)** The p-ERK/ERK ratio in siWT1 groups was reported as fold change compared to siNEG ones (*n* = 3; *P < 0.05 compared to respective siNEG group).

Cell cycle machinery is regulated by pathways that are often defined as driving force of cell survival or death. The expression of downstream molecules of both PI3K/ AKT/mTOR and Raf/MEK/ERK pathways were examined. In particular, pAKT, AKT, pERK, and ERK proteins were studied in WT1-silenced MG63 cells and compared to their own expression in each siNEG group (Figure 6A). At 50 nM p-siWT1 the ratio of pAKT/AKT (Figure 6B) and of pERK/ERK (Figure 6C) reached a significant decrement, while at 25 nM p-siWT1 treatment only the ratio of pAKT/AKT proteins slightly decreased (Figure 6B). These results indicated that downregulation of WT1 protein is linked to alteration of PI3K/AKT pathway, suggesting a positive feedback between AKT phosphorylation and WT1 expression. Considering that each element of these pathways have been shown to participate in G1 progression and to regulate positively cyclin D1 expression, these data fit with the reported analysis of cell cycle markers (Figure 5).

### WT1 silencing induced apoptosis of MG-63 cell lines

**Figure 7:**
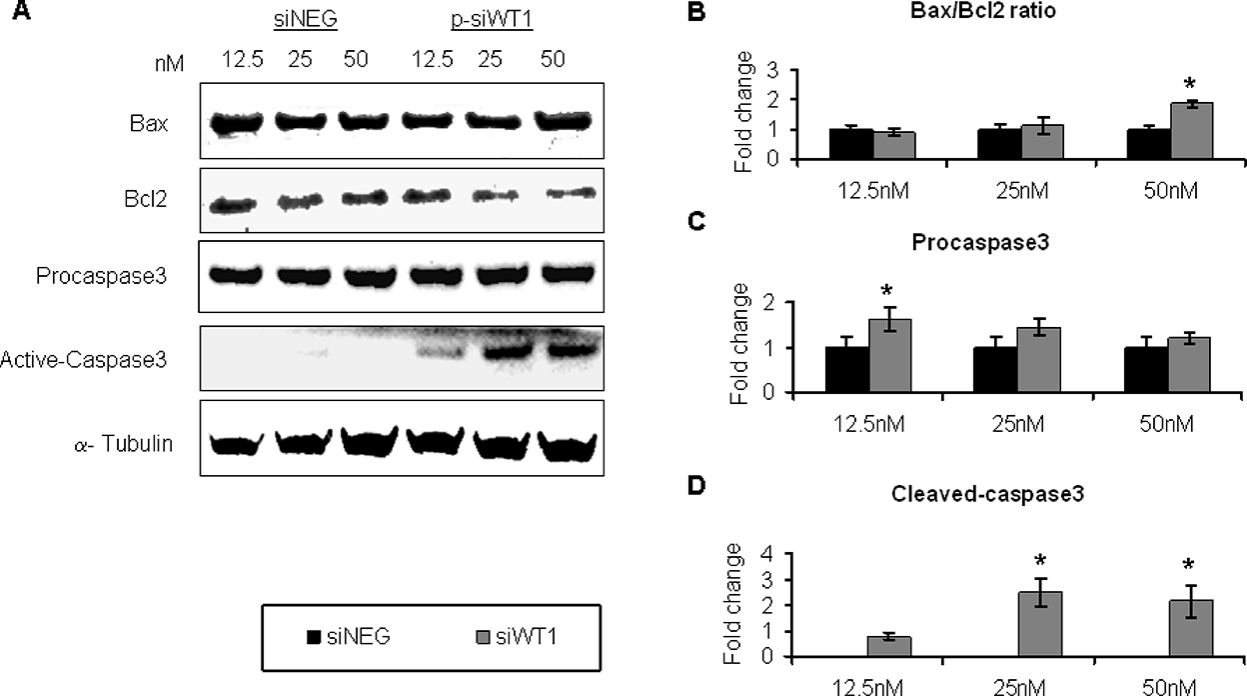
siWT1 induced apoptosis in MG-63 cell lines **(A)** Representative immunoblotting of Bax, Bcl2, procaspase-3, and active-caspase-3 in MG-63 cells treated with 12.5 nM, 25 nM and 50 nM siNEG or p-siWT1. **(B)** The Bax/BCL2 ratio in siWT1 was reported as fold change compared to each siNEG ones (*n* = 3; *P < 0.05 compared to respective siNEG group). Procaspase-3 **(C)**, and active-caspase-3 **(D)** expression in siWT1 MG63 cells was quantified as fold change compared to each siNEG group (*n* = 3; *P < 0.05 compared to respective siNEG group).

The downregulation of cell survival pathways should be associated to programmed cell death machinery activation. Results from cell proliferation and viability could reflect a cytostatic effect and the analysis of trypan blue-stained cells did not give the possibility to evaluate the activation of programmed cell death. Thus, to test the effect of siWT1 on apoptosis, we examined some proteins that act as inducer and effectors of the apoptotic pathway. Specifically, the expression of Bax, Bcl2, procaspase-3, and active-caspase-3 were analyzed by Western blot analysis in MG-63 cells treated with siWT1 and compared to negative control (Figure 7A). A reduction of the antiapoptotic Bcl2 was revealed slightly at 25 nM p-siWT1 treatment, whereas it was significant in 50 nM WT1-silenced MG-63 (Figure 7A). These events turned into an increased Bax/Bcl2 ratio that indicated the induction of apoptotic event (Figure 7B). As effectors of programmed-cell death, procaspase-3 and active-caspase-3 proteins were detected. Results demonstrated that even if the procaspase-3 expression did not change (Figure 7C), its cleaved-active form increased in p-siWT1-silenced cells (Figure 7D). Moreover, the intensity of caspase-3 activation followed the efficiency of interference treatment, as the maximum level was reached at 50 nM p-siWT1 treatment (Figure 7D). Moreover, to investigate autophagic flux in OS cells, we examined the conversion of LC3-I to LC3-II, as an indicator of autophagic activity (Supplementary Figure 2). The results indicated that the expression of WT1 correlated with active autophagy. All siNEG groups expressed a higher level of WT1 and showed autophagy activity (Supplementary Figure 2), while the loss of WT1 by siRNA was linked to weak autophagy indicated by lower conversion or disappearance of LC3-I to LC3-II. This event suggests that autophagy is an important mechanism for MG-63 cell survival in their physiological conditions.

### Alteration of protein profile in WT1-silenced MG-63 cell lines is related to p53

**Figure 8:**
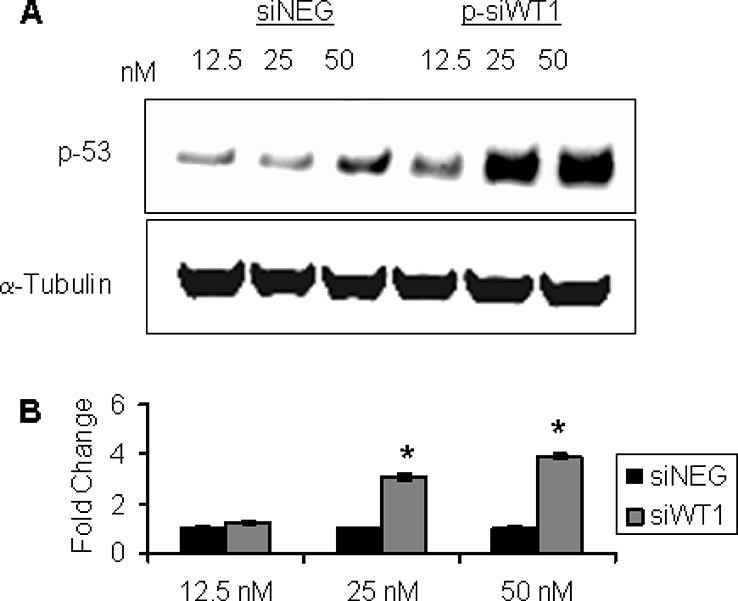
p53 participated to cell death induction by siWT1 in MG-63 cells **(A)** Representative immunoblotting of p53 in MG-63 cells treated with 12.5 nM, 25 nM and 50 nM siNEG or p-siWT1. **(B)** Data represented the average value of three independent transfection experiments ± SEM and siWT1 results were expressed as fold change compared to siNEG ones (*n* = 3; *P < 0.05 compared to respective siNEG group).

Considering that the expression of some previously analyzed proteins is related to the p53 pathway, the analysis of p53 expression profile was performed in WT1-silenced MG-63 cells with respect to siNEG-treated ones (Figure 8A). The level of p53 in the siNEG-treated MG-63 cells did not change significantly, but it increased in p-siWT1 group. In particular, p53 expression was significantly induced in WT1-silenced MG-63 cells with 25 and 50 nM of siRNA pool (Figure 8B).

## DISCUSSION

It is almost well known that WT1 is a multi-facets protein, playing also opposite roles in the same cell population according to the functional context in which it operates [9, 20]. Supported by a variety of evidences, we fully agree with the emerging idea that WT1 functions depend on various factors. Considering the past and the newest data from the literature, it seems increasingly clear that each elucidated piece exposes just the tip of an iceberg that has to be explored, yet.

First of all, the complex role played by WT1 is largely linked to diversified WT1 gene encoding profile. An intricate pattern of mRNA species, a combination of alternative splicing, an alternative translation start sites and RNA editing [9] are fundamental factors that contribute to define the multifaceted and often contradictory function of the protein product of the WT1 gene. Moreover, this profile is dynamically modulated according to the functional context in which WT1 operates. For example, contradicting the traditional idea of neuroblastoma considered as a WT1-negative tumor, we recently evidenced the complex picture of WT1 in this aggressive extracranial pediatric tumor, where specific WT1 isoforms correlate both with oncogenesis of neuroblastoma and cells differentiation state [11]. Also in OS, WT1 identikit appears to be very dynamic. Previous studies strongly indicate that the non-mutated wild-type WT1 gene is involved in tumorigenesis of various types of human bone and soft-tissue sarcomas including OS and that it is expressed at protein level in a larger number of highly proliferating cancer cells [37]. Moreover, a network analysis of the pathobiology of this neoplasm underscored that OS is a contextual result of distinct patterns of interactions between multiple genes via various biological processes and pathways, including regulation of cell apoptosis, proliferation, antigen processing/presentation, and phosphatidylinositol signaling system [47].

Our results indicated that WT1 fits perfectly in the complicated network activated in OS. In fact, as previously reported [23], we firstly confirmed WT1 expression in 50% of human OS cases, suggesting that WT1 expression profile may be dynamically and timely modulated according to the functional and stage-specific contexts of tumorigenesis in which WT1 operates.

Thus, we intended contribute to decode the WT1 involvement in OS by a cancer cell line model that closely mimics the patient derived and WT1-positive OS. The choice of MG-63 cell line is justified by a series of experimental evidences: i) it has an enrichment of WT1 gene respect to normal osteoblasts [44]; ii) it has been recognized to possess the typical complex genomic structure that characterizes patient derived OSs [45]; iii) it has been already reported as a good model for colony forming ability, proliferation and tumorigenesis functional assays [45]. Moreover, we intentionally avoided the use of WT1-inducible system and we preferred an already established WT1-positive tumor cell line because the applicable over-expressing system may reflect the function of WT1 only to a limited extent. By analyzing the WT1 profile in MG-63 cell line, we found a predominant expression in the cytoplasmic area around the nucleus and a weaker, but constant, distribution in the nuclear zone. This localization is in line with the complex role played by WT1 in diverse cellular activities and the modulation of many genes and cofactors including i) interaction with actin as a specific adaptor protein in carrying on the cytoskeletal changes activated in cancer cells in both nucleus and cytoplasm [48], ii) relationship with p53 and STAT3 in carrying on influence of target genes triggering cell proliferation [49, 50], iii) activity in nuclear-cytoplasmic shuttling under appropriate conditions consistent with its high expression level in the perinuclear zone of MG-63 cells [51].

We then demonstrated that an efficient WT1 targeting siRNA suppresses cell proliferation, induces cell cycle arrest and activates apoptosis in MG-63 cell line. Cyclin D1, cyclin E, CdK4, CdK1/2, and cell cycle inhibitors Rb and p27 proteins, some of which has been already reported as G1 phase regulators in MG-63 OS cell line [52], were affected by WT1 down-regulation.

Significant are the pRb and p53 results, which were down- and up-regulated, respectively. As well known, pRB and p53 pathways play a significant role in the development of most human cancers [53]. pRb phosphorylation seems to be related to mitogen signals, which converge on the cell cycle machinery, represented by the cyclin D1/CdK4 complex in the early and mid-G1, and cyclin E/CdK2 in late G1 [53–54]. An interesting study demonstrated that osteoblasts depend on both pRb and cell-to-cell contacts for their differentiation and function [55] and both adherent junction defect at osteoblast membranes and pRb inactivation contribute to OS formation in Rb knock-out mice [55]. In our work, the high reduction of phosphorylated Rb, together with the results of cyclins/CdK complexes and p27, strongly suggest a WT1 contribution in cell proliferation.

Also noteworthy is the up-regulation of p53 showed in WT1-suppressed MG-63 cells. Tumor suppressor pathway governed by p53 gene are known to be involved in the pathogenesis of OS [56]. Previous studies have presented evidence for a protein-protein interaction between WT1 and p53 in baby rat kidney cells [57] and Wilms’ tumors [58] as well as a WT1-induced p53 protein stabilization in Saos-2 cells [59]. Our results, showing a direct connection between p53 pathway and WT1 purview, suggest a WT1 contribution to p53 central role in monitoring cell growth, cell cycle and its regulation, DNA damage repair and induction of apoptosis [60–62].

Finally, we also found that PI3K/AKT/mTOR and Raf/MEK/ERK signaling pathways decreased due to loss of WT1, suggesting the proliferative effect of WT1 also in MG-63 OS cells by modulating signal transduction according to previous studies [47, 63–66].

Our results showed that also apoptotic machinery is strongly influenced following to the interference effect of siWT1 in MG-63. Our data seem to contrast other previous reports about WT1 role in other OS cell lines. In fact, WT1 ectopic and inducible expression has been associated to apoptosis induction in the U2OS and Saos-2 OS cell lines [67–70] and WT1 isoforms lacking the KTS insert have been associated to activation of the bcl-2 promoter in transiently transfected Saos-2 [71]. Thus, it should be stressed that each hypothesis and conclusion should be tested both in specific functional context in which WT1 operates and in agreement with the physiology of applied cell lines. In fact, U2OS and Saos-2 cells are recognized as WT1-inducible systems, while it has been recently elucidated that MG-63 cells express a higher and stable basal level of WT1 associated with a more autophagy activity than U2OS and Saos-2 cells [72]. It has been suggested that MG-63 maintain their own homeostasis by autophagy. Because autophagy promotes the protection of cells from apoptosis [66, 73], this feature may be associated with the carcinogenesis of OS. In our experimental model, the suppression of WT1 by silencing RNA turned into homeostasis unbalance and sensitized MG-63 to undergo to apoptosis. Reduced Bcl-2 protein expression, appearance of cleaved caspase-3, upregulation of p53, and enhancing Bax, observed after WT1 silencing, are consistent with an oncogenic role of WT1, similarly to other investigated cell type [74–76], and in opposition to others, where WT1 could suppress Bcl-2 family protein functioning as a tumor suppressor [77].

In conclusion, our work strongly confirmed WT1 expression in human OS tissues and its ability to operate in cellular fate. Even if the regulation of WT1 and its effects on cell proliferation, cell cycle, and apoptosis seem to be cell-type and context specific, this study suggests that WT1 is an oncogene in OS via multiple pathways. Thus, putting light on the complex WT1 interaction network could, piece by piece, provide the tools to counteract with a greater chance of success WT1-positive OS.

## MATERIALS AND METHODS

### Human tissues

Biopsies of primary osteogenic sarcoma of the bone were obtained from 6 patients with an age ranging from 9 to 68 years. The cases were retrieved from the pathology files of the Anatomic Pathology Section of Department of Medical and Surgical Sciences and Advanced Technologies G.F. Ingrassia, University of Catania. Six cases of conventional high grade osteogenic sarcoma were collected. For each single case, at least one paraffin block was available. All the Haematoxylin and Eosin slides were reviewed and the diagnoses were confirmed.

### Immunohistochemistry

Immunohistochemical analyses were performed using the standard streptavidin–biotin labeling technique using the LSAB kit (Dako, Glostrup, Denmark) with appropriate positive and negative controls. Sections derived from paraffin embedded specimens were deparaffinized in xylene for 15 min, rehydrated, and treated with 3% H_2_O_2_ for 10 min to block endogenous peroxidase activity, followed by extensive rinsing in double-distilled water and further rinsing for 15 min in 0.01 M phosphate-buffered saline (PBS), pH 7.4. Deparaffinized sections were incubated with anti-WT1 antibodies (clone 6F-H2, anti-N terminus, from Dako Glostrup Denmark; clone C-19, anti-C terminus from Santa Cruz Biotechnology). Microwave pre-treatment was crucial to enhance the staining in all samples examined. Accordingly, all sections were pre-treated with citrate buffer (pH 6.0) and exposed to radiation in a microwave oven. To reduce the commonly seen non-specific immunoreactivity due to endogenous biotin, sections were pre-treated with 10 mg/mL of ovalbumin in PBS followed by 0.2% biotin in PBS, each for 15 min at room temperature. Bound antibody was revealed by incubation with 3,3-diaminobenzidine (Sigma–Aldrich, St. Louis, MO, USA) in 0.01% H_2_O_2_ for 15 min at room temperature. Sections were then counterstained with Haematoxylin, dehydrated, and mounted. Negative controls involving the omission of the primary antibody were included. With regard to WT1 immunostaining, both cytoplasmic and nuclear immunoreactivity were evaluated. The percentage of positively stained cells was assessed by semi-quantitative optical analysis according to a four-tiered system (< 1% positive cells = negative staining; 1–10% positive cells = focal staining; 11–50% positive cells = heterogeneous staining; > 50% positive cells = diffuse staining). Staining intensity was graded into weak, moderate, or strong.

### Cell culture

Human OS cell line, MG-63, was obtained from American Type Culture Collection (ATCC). Cell line was cultured in Eagle's Minimum Essential Medium (EMEM) containing 10% heat-inactivated fetal bovine serum (FBS) (Invitrogen, Carlsbad, CA, USA). Cells were maintained in a humidified 37°C incubator with 5% CO_2_. The medium was changed two times per week and a subcultivation ratio of 1:4 was performed. The cells were passaged twice a week up to passage 10 after being thawed from stock culture.

### Immunocytochemistry

For immunofluorescence assays, MG-63 cells were fixed with 4% formaldehyde for 15 min, permeabilized with 0.1% Triton X-100 for 10 min, and washed with PBS. After blocking with 5% normal goat serum in PBS-Triton X-100 cells were incubated overnight at 4°C with the following primary antibodies against WT1: C-19 (sc-192, anti-C terminus from Santa Cruz Biotechnology, Inc.) and clone 6F-H2 (anti-N terminus, from Millipore). Cells were then washed three times with PBS for 5 min, followed by 1 hour incubation with the appropriate fluorescent dye-conjugated secondary antibody.

Chromosomal DNA was stained with DAPI and imaging was performed with a Leica fluorescence microscope. Negative controls involving the omission of the primary antibody were included. Image analysis was performed with Leica Application Suite software.

### Subcellular fractionation

Cells were detached by trypsin-EDTA, washed three times with ice-cold PBS and collected in microcentrifuge tubes by centrifugation at 3300 r.p.m. for 10 min at 4°C. The whole proteins were extracted with lysis buffer (10 mM Tris–HCl plus 10 mM KCl, 2 mM MgCl_2_, 0.6 mM PMSF, and 1% SDS, pH 7.4) enriched with Protease and Phosphatase Inhibitor Cocktail Tablets (Roche Applied Science). For the subcellular fractionation, aliquots of 1 × 10^6^ cells were suspended in 150 μl of buffer A (10 mM Hepes, pH 7.9, 1.5 mM MgCl_2_, 10 mM KCl, 0.5 mM dithiothreitol, 0.2 mM phenylmethylsulfonylfluoride), incubated on ice for 15 min and homogenized by 15 passages through a 25-gauge needle, followed by centrifugation at 12000 r.p.m. for 40 s at 4°C. The supernatants were collected and stored as a cytoplasmic fraction, whereas the pelleted nuclei were washed in 70 μl of buffer A and re-suspended in buffer B (20 mM Hepes, pH 7.9, 25% glycerol, 0.42 M NaCl, 1.5 mM MgCl_2_, 0.2 mM EDTA, 0.5 mM dithiothreitol, 0.5 mM phenylmethylsulfonylfluoride) supplemented with 1× of protease inhibitor cocktail (Roche Applied Science). After 30 min incubation on ice, the nuclear extracts were collected by centrifugation at 12000 r.p.m. for 5 min. The extracts were rapidly frozen and stored at –80°C until processed for Western blot. Before freezing, the protein concentration was estimated using the Bicinchoninic acid assay (Pierce).

### siRNA transfection of MG-63 cell line

A total of 5 × 10^4^ cells were seeded into each well of a 6-well tissue plate. The next day, 40–50% confluence, the cells were transfected with siRNA against WT1 (siRNA-WT1) (Invitrogen Milan, Italy) using transfection reagent, Lipofectamine 2000, at a final concentration of 0.2%. As previously reported [36], siRNA-WT1 consisted of one (s-siWT1) sequence, 5′-AAAUAUCUCUUA UUGCAGCCUGGGU-3′ (WT1-HSS111388), or a pool (p-siWT1) of the following sequences: (WT1-HSS111388), 5′-UUAAGGUGGCUCCUAAGUUCAUCUG-3′ (WT1- HSS187705), 5′-UUUCACACCUGUAUGUCUCCUU UGG-3′ (WT1-HSS111390) and a scrambled stealth RNAi oligonucleotide was used as a control (Invitrogen). All procedures were performed in an RNase-free environment. To minimize the cytotoxicity of the reagent itself, cells were washed with medium without FBS and antibiotics. Cells were harvested 48 hours after transfection with different concentrations of siRNA (12.5, 25 and 50 nM).

### Western blot analysis

Western blot analysis was performed as already reported in [78]. Equal amount of proteins was boiled in LDS sample buffer (Invitrogen) in presence of 1× sample reducing agent (Invitrogen). Each sample was then subjected to electrophoresis on Bolt™ 4–12% Bis-Tris Plus Gels (Invitrogen). After electrophoresis, proteins were transferred to nitrocellulose membrane, in a wet system, and proteins transfer was verified by staining membranes with Ponceau S. Membranes were blocked with Tris buffered saline containing 0.01% Tween-20 (TBST) and 5% non-fat dry milk for 1 hour, and then probed overnight at 4°C with the following primary antibodies: anti-WT1 (C-19:sc-192, Santa Cruz Biotechnology, 1:200), -Cdk1/Cdk2 (AN21.2:sc-53219, Santa Cruz Biotechnology, 1:200), -Cdk4 (DSC-35:sc-23896, Santa Cruz Biotechnology, 1:200), -cyclin D1 (M3642, Dako, 1:500), -cyclin E (M20:sc-481, Santa Cruz Biotechnology, 1:200), -p27 (3698, Cell Signaling, 1:1000), -p-pRb (KAM-CP121, Enzo Life Sciences, 1:1000), -AKT (9272, Cell Signaling, 1:1000), -pAKT (4058, Cell Signaling, 1:1000), -pERK (9101, Cell Signaling, 1:1000), -ERK (MAB1576, R&D Systems, 1:1000), -Bax (B3428, Sigma-Aldrich, 1:1000), -Bcl2 (SAB2500154, Sigma-Aldrich, 1:500), -caspase-3 (H227:sc-7148, Santa Cruz Biotechnology, 1:200), -caspase-3, Active (C8487, Sigma-Aldrich, 1:500), -p53 (P6749, Sigma-Aldrich, 1:1000), -α- Tubulin (T9026, Sigma-Aldrich, 1:5000), -lamin A/C (N-18:sc6215, Santa Cruz Biotechnology, 1:200). The membranes were rinsed three times in TBST and the appropriate HRP-conjugated secondary antibody was incubated for 1 hour at RT (sc-2030, goat anti-rabbit, 1:20000; sc-2005, goat anti mouse, 1:5000; sc-2020, donkey anti-goat, 1:5000, all from Santa Cruz Biotechnology). The blots were developed using enhanced chemiluminescent solution (Millipore) and visualized with a chemiluminescent Western blot imaging systems (Alliance, UVITEC). Bands were measured densitometrically and their relative density was calculated based on the density of the -a tubulin or lamin A/C signals in each sample. For extracts from subcellular fractionation, values were expressed as arbitrary densitometric units (A.D.U.) corresponding to signal intensity, while siRNA results were reported as protein fold change vs scrambled controls.

### Cell proliferation assay

After transfection, siRNA-transfected cells were harvested at specific time points. The total viable cell number was assessed by trypan blue exclusion assay and counted by a hemocytometer under an inverted microscope (Leica).

### Cell viability assay

The effects of WT1 silencing on cell viability and mitochondrial activity were evaluated with a test based on the cleavage of 3-(4,5-dimethylthiazol-2-yl)-2,5-diphenyltetrazolium bromide (MTT) by mitochondrial dehydrogenases of metabolically active cells. Briefly, the cells were plated in 96-well tissue culture plates at a density of 1 × 10^4^ cells per well and allowed to attach overnight, cells were transfected with si-WT1 for 48 hours, and then incubated with MTT (20 μl of 5 mg/mL PBS) for 3 hours. The formazan precipitate was dissolved in 200 μl of dimethylsulfoxide (DMSO). The absorbance at 550 nm was measured by a benchmark microplate reader (Bio-Rad, Hercules, CA) and results reported as percentage respect to controls.

### Statistical analysis

In this study, the results are expressed as the means ± SEM. All experiments were repeated at least three times. Statistical significance was determined by Student's *t-test*, and *P*-values < 0.05 were considered to indicate statistically significant differences.

### Abbreviations

OS, osteosarcoma; WT1, Wilms’ Tumor Gene 1; siRNA, silencing RNA; CdK, cyclin-dependent kinase; PI3K, phosphatidylinositol 3-kinase; mTOR, target of rapamycin; ERK, extracellular signal-regulated kinase.

## SUPPLEMENTARY MATERIALS FIGURES AND TABLES


