# Supplementary material for: Wilms' tumor gene 1 silencing inhibits proliferation of human osteosarcoma MG-63 cell line by cell cycle arrest and apoptosis activation
Source: Oncotarget. 2017 Jan 18;8(8):13917–31. doi: 10.18632/oncotarget.14715 (PMC5355150; doi:10.18632/oncotarget.14715)
Supplement: Supplementary file 1 [file oncotarget-08-13917-s001.pdf]

Wilms' tumor gene 1 silencing inhibits proliferation of human osteosarcoma MG-63 cell line by cell cycle arrest and apoptosis activation

Supplementary Materials

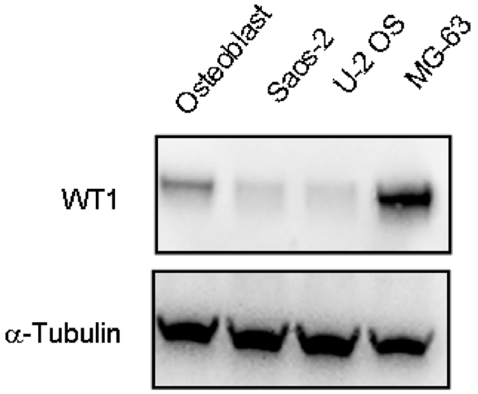

Supplementary Figure 1: WT1 expression in different cell lines. Protein levels of WT1 in Osteoblast, Saos-2, U-2 OS and MG-63 cells.

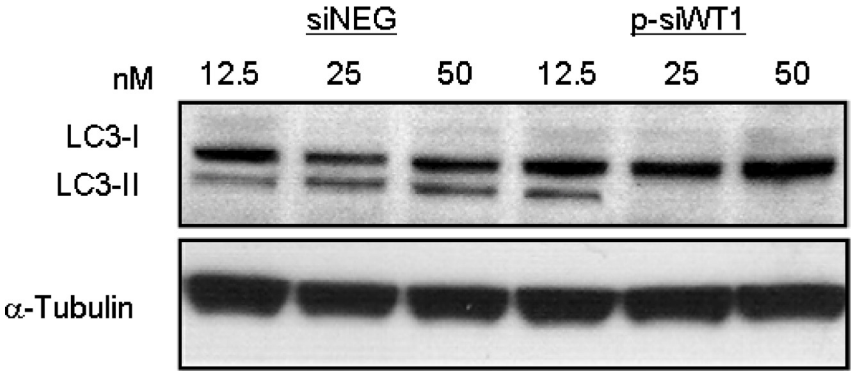

Supplementary Figure 2: WT1 knockdown reduced LC3-II level. Representative Immunoblotting of LC3-I and LC3-II in MG-63 cells treated with 12.5 nM, 25 nM and 50 nM siNEG or p-siWT1.
